# Food Neophobia and Consumer Choices within Vietnamese Menu in a Polish Cohort Study

**DOI:** 10.3390/ijerph18062925

**Published:** 2021-03-12

**Authors:** Dominika Guzek, Duy Nguyen, Dominika Głąbska

**Affiliations:** 1 Department of Food Market and Consumer Research, Institute of Human Nutrition Sciences, Warsaw University of Life Sciences (WULS-SGGW), 159C Nowoursynowska Street, 02-776 Warsaw, Poland; duy.nguyen.sggw@gmail.com; 2 Department of Dietetics, Institute of Human Nutrition Sciences, Warsaw University of Life Sciences (WULS-SGGW), 159C Nowoursynowska Street, 02-776 Warsaw, Poland; dominika_glabska@sggw.edu.pl

**Keywords:** food neophobia scale, choice, consumer, ingredients, menu, Vietnamese cuisine

## Abstract

One of the factors influencing consumer food choices is food neophobia (FN), described as a reluctance to try novel or unknown food products. The aim of the study was to determine the influence of FN on food choices in young Polish respondents through a web-based choice experiment with Vietnamese restaurant menu. The choice experiment was conducted using a Computer-Assisted Web Interview (CAWI) method in a sample of 601 young adults, while using a developed Vietnamese restaurant menu. For the dishes, neophobic potential for a Polish population was defined, based on content of ingredients not typical for Polish diet. The FN was assessed using the Food Neophobia Scale (FNS) by Pliner and Hobden. The neophobic potential was the determinant of choice of dishes (*p* < 0.05). The participants characterized by a high FN level less commonly than others chosen dishes characterized by neophobic potential as: starter (Nem quõn—non-fried spring rolls with shrimps) (*p* = 0.0003), soup (Mién gà—soup with cellophane noodles and nam huong mushrooms) (*p* < 0.0001), main course (Phở xào bò—rice noodles with soy sauce and fish sauce) (*p* < 0.0001) and dessert (Chè thập cãm—dessert of golden gram, black eye beans, Azuki beans and tapioca) than other options (*p* = 0.0007). It was stated that FN in young respondents may reduce the frequency of choosing dishes containing unfamiliar ingredients and, as a result, it may cause lower diversity of consumed dishes. Taking into account that not properly balanced diets resulting from rejecting some types of products are becoming a growing problem, the FN should be taken into account in the general public health policy.

## 1. Introduction

The nutritional behaviors are determined by numerous biological, anthropological, economic, psychological, socio-cultural and home-related factors [1], while their influences are competing, reinforcing and interacting [2], as well as they are shaped by the individual situation [1]. Among the factors important for adults, there are those associated with socio-demographic attributes, but not only those factors should be taken into account, as the other determinants may be even more influent [3]. Recently, increased attention has been paid to the role of psychological factors, including personality or experience, as food is no longer a basic need, but it is also as a source of pleasure, socialization and cultural transmission [4]. The other factors of a great influence on the food choices are associated with health and environmental aspects [5], as well as placement, advertising and novelty [6]. However, regardless of knowledge of these factors, the food choices determinants are still not fully understood and more research is needed to define possibility to influence nutritional behaviors and promote beneficial ones [7].

A recent systematic review of Torres et al. [8] presenting the issue of food neophobia (FN) in children, emphasized the role of this factor as one of the most significant determinants of nutritional behaviors. The FN may be studied while using the Food Neophobia Scale (FNS) by Pliner and Hobden [9], which is commonly applied, as it has been translated into various languages. The FNS allows to attribute for each respondent specific FN category, which is associated with willingness to try or to eat unfamiliar foods [10] or ethnic foods [11], but also with not liking such foods [12].

Especially in childhood this factor is crucial, as in this specific period the future eating habits are shaped [13]. However, it is indicated that the FN level developed in childhood may be fairly stable thought life [14] or even may be increased in older people [10]. The FN is defined as avoidance of, or reluctance to try unfamiliar or novel foods that may cause even rejection of some products [15]. Taking into account the nutritional value of diet that is followed, it may be a significant problem, due to the fact, that it reduces the intake of specific products or even groups of products, so may cause lack of diversity and reduce intake of essential nutrients [16]. Moreover, in case of individuals responsible for food purchase decisions in household, it may also influence willingness to pay and purchase intentions [17].

Regardless of the causes and factors influencing the FN level in children, adolescences and adults, it may negatively influence not only the variety of the food products in diet, but mainly the general quality of the diet [9,18]. In the study of Cooke et al. [19], which was associated with the predictors of fruit and vegetable consumption in 2–6 years old British children, authors stated that children who were more neophobic ate less often fruit and vegetables than children who were less neophobic. Similarly, in the previous Polish study, which was conducted in a group of children aged 10–12, children who were more neophobic ate less often vegetables than children who were less neophobic [16]. In adults, such observation also was indicated for vegetable, which supports the hypothesis that FN may have similar effects on children and adults, as well as that level of FN and its influence on food choices could remain stable from childhood until adulthood [14].

In general, FN is significantly associated with food choices [20] namely with how individuals decide on what they want to eat [21]. The results of the previous studies indicated that FN level influences the food products choices not only during grocery shopping [22], but also while eating at the restaurant serving dishes of familiar cuisine [23]. However, FN appears to be a very complex attitude that may be modulated by a number of other factors [24], while only few studies have examined influence of FN on everyday food choices [25,26]. Based on the presented background, the aim of the study was to determine the influence of FN on the food choices in the Polish young individuals through a web-based choice experiment with Vietnamese restaurant menu (being for general Polish consumers unfamiliar cuisine).

## 2. Materials and Methods

### 2.1. Study Design

The Choice Experiment (CE) method was selected to assess the food choices in the studied group, as this method is used to analyze the choices when individuals are asked to select their preferred alternative from a set of alternatives, while: (1) alternatives can be described by their attributes, (2) an individual’s valuation depends upon the levels of these attributes and (3) choices are based on a latent utility function [27]. The CE method is widely used except for the marketing studies, also in the nutritional studies to evaluate individual preferences that influence meal choices [28].

### 2.2. Participants

The study was conducted while using a Computer-Assisted Web Interview (CAWI) to recruit the participants, while the information about the study was disseminated via social media.

The inclusion criteria were as follows: men and women, young adults (aged 18–30 years) and Caucasian individuals of the Polish ethnicity. The exclusion criteria were as follows: declared food allergy (either diagnosed by physician, or based on any symptoms), any missing data in the provided questionnaire and no informed consent to participate. Only food allergies were taken into account during the recruitment procedure and no other concurrent diseases were included. Moreover, no additional variables were taken into account during recruitment to obtain the studied group of diverse characteristics.

### 2.3. Choice Experiment (CE)

The CE was conducted based on the mock Vietnamese model restaurant menu, which was developed for this experiment by the Vietnamese ethnicity chef, Polish-speaking and living in Poland, with nutritional knowledge and being familiar with the concept of FN. Afterwards, the dishes were verified by Polish nutritionists, taking into account understandability for Polish consumers and the neophobic potential of dishes, while they were discussed and corrected, if needed.

The Vietnamese menu was chosen as an example of cuisine being for general Polish consumers unfamiliar. The comparison of Polish and Vietnamese culture reveals a number of differences [29] and Vietnamese cuisine is not indicated by Polish consumers among the most preferred ones [30]. In spite of the fact that in Poland restaurants run by Vietnamese owners exist since 1990s, they serve mainly inexpensive fast food, being rather general Asian cuisine, or some other Asian ethnic cuisines, prepared in such way to be accepted by Polish consumers [31].

The mock Vietnamese model restaurant menu was developed to include dishes in 4 categories: starter, soup, main course and dessert, in such way, to present in each category 3 various Vietnamese dishes (of a comparable energy value), including one dish in each category that may be defined as one with neophobic potential for a Polish population. The neophobic potential was based on the content of ingredients unusual for typical Polish diet (e.g., shrimps, cellophane noodles), using not typical ingredients for a specific dish (e.g., omelet in spring-rolls, dessert based on beans), or preparation (e.g., non-fried spring-rolls, dessert served in layers in a glass). Each dish was presented with its name in Vietnamese and a short description in Polish, while the menu was not branded with any restaurant name and it presented dishes with no prices, photographs or any information other than those needed for the CE and was as simple as possible.

The dishes included to the developed Vietnamese model restaurant menu with their descriptions and defined neophobic potential are described in Table 1.

Each respondent was asked to choose dishes which they would order while being in Vietnamese restaurant. They were instructed to not take into account the supposed prices of dishes and to choose whatever they would like to. They were encouraged to choose one dish in each category of starter, soup, main course and dessert, but they were allowed to indicate that they do not want to choose any of the dishes presented in category (if while being in restaurant, they would not choose any of the dishes presented).

### 2.4. Measures

#### 2.4.1. Choice of Dishes within CE

The major variable in the study was the choice of dishes, which was interpreted in the context of the research question associated with the influence of FN level on the choice of dishes.

#### 2.4.2. Food Neophobia (FN) Level

The FN level was assessed based on the FNS questionnaire by Pliner and Hobden [9], which constitutes a 10-items scale with statements to be rated by a respondent in a 7-points Likert scale (with 1 referred as strongly disagree and 7 referred as strongly agree). As 5 of the items are positive ones and 5 are negative ones (while agreed, indicting either FN or lack of FN), for interpretation, answers to negative ones were to be reversed. Afterwards, the answers were to be attributed for each item to points, resulting in the total score (from 10 to 70), that may be interpreted within FN categories. The FN categories are based on the score interpreted within the studied group, with cut-offs based on terciles, as analyzed by other authors [32]. The following groups were determined:

(1) Low FN level—first tercile of FNS score (values of 10–26) (*n* = 200),
(2) Average FN level—second tercile of FNS score (values of 26–36) (*n* = 201),
(3) High FN level—third tercile of FNS score (values of 36–61) (*n* = 200).

#### 2.4.3. Other Characteristics of the Studied Group

The additional variables which were assessed in the studied group included socio-demographic characteristics: sex (to choose: men, women), age (open-ended question), place of residence (to choose: village; towns and cities of <500,000 residents; cities of >500,000 residents), as well as economic status (subjectively described as: very bad or bad; average; good or very good). The question about economic status was not compulsory and respondents were allowed to not answer this question.

The respondents were also asked about their general approach towards Vietnamese cuisine, with simple statements to be rated by a respondent in a 7-point Likert scale (from strongly disagree to strongly agree). The following statements were presented:

(1) I would like to go to restaurants serving Asian ethnic cuisines.
(2) I would try a dish of not attractive appearance.
(3) I would try a dish of unpleasant smell.
(4) I would like to learn to cook Vietnamese dishes.
(5) While ordering a dish I take into account information about allergens.

Moreover, they were asked about hunger sensation while participating in the study to verify if it may interfere with their responses. Similarly, as for questions listed above, they received a simple statement (I am hungry now), to be rated in a 7-point Likert scale (from strongly disagree to strongly agree).

#### 2.4.4. Characteristics of the Sample

The characteristics of the participants of the study is described in Table 2. The majority of respondents were 18–21 years (over 75%), women (over 70%) and living in big cities (over 50%), as well as they declared good or very good economic status (over 50%).

### 2.5. Statistical Analysis

The results are described as means ± standard deviation (SD) with median, 25th and 75th values, while distributions were verified using the Shapiro–Wilk test. For the comparison of groups, chi^2^ test was applied.

The construct validity of the FNS was assessed by the Confirmatory Factor Analysis (CFA). To verify the results, the overall fit of the models was assessed while using the Comparative Fit Index (CFI), Standardized Root-Mean Square Residual (SRMR) and Root Mean Square Error of Approximation (RMSEA). CFI of Diagonally Weighted Least Squares (DWLS) was calculated, as DWLS provide more reliable model inference with small-to-medium sample sizes and ordinal data [33]. The following cutoff criteria were applied: value of 0.90 or more for CFI [34]; value of 0.08 or less for SRMR [33] and value of 0.06 or less for RMSEA [35]. The CFA presented good model fit, with χ^2^ = 132.425 (degrees of freedom [df] = 34), CFI = 0.968, RMSEA = 0.069 (90% confidence interval [CI: 0.057, 0.082]) and SRMR = 0.06.

The internal consistency in case of the FNS was analyzed using the Cronbach’s alpha coefficient (α) and the cutoff criteria of 0.70 or more was applied [36]. In the presented study the Cronbach’s alpha was on the 0.76, indicating good internal consistency.

The accepted level of significance was set at *p* ≤ 0.05. Statistical analysis was conducted using Statistica software version 13.3 (TIBCO Software Inc., Palo Alto, CA, USA) and JASP 0.14.0.0 (JASP Team 2020, University of Amsterdam, Amsterdam, The Netherlands).

## 3. Results

The FNS scores of the participants are presented in Table 3. The FNS scores ranged from 10 to 61, while the possible range is 10–70. For total studied group, the median score was 31 (non-parametric distribution of data) and for the low, average and high FN level respondents, the median scores were 20, 31 and 41, respectively.

The comparison of the general approach towards Vietnamese cuisine and towards information about allergens, accompanied by hunger sensation stratified by FN level of participants is presented in Table 4. It was observed that participants with high FN level less likely declared that they would like to go the restaurants serving Asian ethnic cuisine, than participants with average and low FN level (*p* < 0.0001). Similarly, regardless of the fact, that most of the respondents declared low or moderate willingness to try dishes characterized by not attractive appearance or unpleasant smell, it was observed, that participants with high FN level declared even lower willingness to try such type of dishes than respondents of the lower FN level (*p* < 0.0001 and *p* < 0.0001, respectively). At the same time, respondents of the lower FN level more often declared that they would like to learn to cook Vietnamese dishes than respondents of the high FN level (*p* < 0.0001). Those differences were observed in the studied group, in spite of the fact that the approach towards information about allergens in dishes (*p* = 0.2795) and level of hunger sensation while participating in the study did not differ (*p* = 0.6293).

The comparison of the choices of dishes from the Vietnamese model restaurant menu, stratified by FN level of participants are described in Table 5. For all the courses, the FN level influenced choices of dishes. In the case of the high FN level, for each course, there was the highest share of participants declaring that they would not choose any of the dishes presented (24.5%, 19.5%, 12.0%, 22.0% for starters, soups, main courses and desserts, respectively), while compared with other FN groups. For each course, the participants with high FN level presented diversified frequency of choosing specific dishes. They less commonly than other dishes chosen those which were defined in CE as ones of neophobic potential, namely Nem quõn, as a starter (*p* = 0.0003), Mién gà, as a soup (*p* < 0.0001), Phở xào bò, as a main course (*p* < 0.0001) and Chè thập cãm, as a dessert (*p* = 0.0007).

## 4. Discussion

The key finding from the conducted research is associated with the fact that neophobic respondents did not choose dishes attributed to neophobic potential as a starter (Nem quõn—non-fried spring rolls with shrimps), soup (Mién gà—soup with cellophane noodles and nam huong mushrooms), main course (Phở xào bò—rice noodles with soy sauce and fish sauce) and dessert (Chè thập cãm—dessert of golden gram, black eye beans, Azuki beans and tapioca) than other options characterized by less neophobic ingredients. Moreover, in the case of the high FN level, for each course, there was the highest share of participants declaring that they would not choose any of the dishes presented, while compared with other FN groups. Taking this into account, it may be stated that, regardless the fact, that for participants of this study, the Vietnamese cuisine and the names of Vietnamese dishes were unfamiliar, there were some neophobic individuals rejecting specific dishes, or even all the dishes. It may have resulted from the individual ingredients of the dishes (shrimps, water cress, cellophane noodles, nam huong mushrooms, fish sauce, black eye beans, Azuki beans, tapioca), fact that ingredients were not typical for a specific dish (omelet in spring-rolls, nuts in main course), or applied preparation (non-fried spring-rolls, dessert served in layers in a glass), that may have an impact on the choice at the time of making the decision.

The observed rejection of novel dishes is a result of FN level, which causes hesitancy to try novel foods, as a potential strategy to protect from poisoning [37]. It should be mentioned that such rejection of food mainly does not occur when individuals are tasting the food products, but it is more often stated during visual observation before eating, as rejection is based only on the basis of the available information such as visual traits, odor, list of ingredients, etc. [38]. It results from the fact that food likability is stated to be related to the clarity of mental imagery for taste, which is based on the visual categorization or likeliness of foods [39]. In spite of the fact, that exposure on visual traits from food may decrease FN level, this observation was formulated for children and not for adults [40]. Moreover, the influence of visual traits on food consumption is related to the visual appeal of the food [40].

In the presented study, the individuals characterized by higher FN level more often, than individuals characterized by average and low FN level, declared that they would not try a dish of not attractive appearance, as well as would not try a dish of unpleasant smell. The observations associated with smell are consistent with the observations of Raudenbush et al. [41], as they stated that individuals of high FN level have been found to rate odors as less pleasant and sniff odor samples less vigorously than those of low FN level. Such situation could be related to the psychological comfort, as in the recent systematic review of Torres et al. [8], authors indicated that all actions associated with the emotional support and comfortable environment can stimulate curiosity about new shapes, colors and texture in children, which may be associated with their FN level. As a consequence, psychological comfort could reduce the level of neophobic behaviors and may contribute to increased positive experiences with food. However, in the presented study, it was observed that the problem may be associated with the fact that individuals of a high FN level do not want to try novel foods and are not interested in unknown cuisine, so it may prevent them from breaking down their FN.

While analyzed the FN level in the studied group, it should be compared with the level observed in the other populations of adults. In the present study, the mean of FN score was 31.0; however, it should be mentioned that the range differs in various countries. The mean of FN score for adults from Canada was 29.6 [42], Denmark—30.1 [43], Belgium—30.6 [44], Korea—33.0 [45], Finland—33.9 [10] or 38.0 [46] and Australia—34.7 [47]. Taking this into account, it may be indicated that the observed level is similar as in other European countries—Denmark and Belgium, which may be associated with the common or similar culture, tradition and food products assortment.

The influence of FN on the food products choices may depend on the type of products; as such, the impact was observed for the products of animal origin, but not for starchy products, sweets, or fatty snack foods [48]. However, as indicated by Helland et al. [49], there are only few studies addressing the associations between FN and meat, as well as one study addressing the associations between FN and fish consumption. Such association between FN level and meat or fish consumption, may be also indicated in the presented own study, as participants of the high FN level rejected some dishes based on the content of seafood and fish, as Nem quõn—non-fried spring rolls with shrimps and Phở xào bò—rice noodles with soy sauce and fish sauce. However, there were also other dishes with a neophobic potential which were rejected based on the content of different ingredients, namely Mién gà—soup with cellophane noodles and nam huong mushrooms, as well as Chè thập cãm—dessert of golden gram, black eye beans, Azuki beans and tapioca.

While assessing approach towards Vietnamese cuisine in a population of Polish consumers, it should be indicated that differences between Polish and Vietnamese cuisines are significant, not only because of the applied ingredients and techniques, but also based on the share of macronutrients. When analyzing the food consumption patterns among Vietnamese, it should be indicated that the main source of energy and protein are rice and other starchy products, followed by meat and meat products [50]. At the same time, in the Polish diet, meat and meat products may be the main food source of protein [51]. Such differences may also influence the general perception of dishes and the fact that some of them may be rejected as unfamiliar. However, the recent study by Anjos et al. [52], conducted in Brazil, indicted that preschoolers with high FN level have lower adherence to traditional dietary patterns than others, so it can be hypothesized, that they refuse to try some unfamiliar products even if such products are typical for dietary pattern in their country. This conclusion is in agreement with the observations from the study of Costa et al. [53], who stated that several traditional Portuguese dishes may be rejected by a neophobic individuals, as those dishes are prepared with animal’s blood, which may result from the fact, that the familiarity of food should be perceived rather as based on individual experience than general cultural approach.

In the study of Raudenbush and Frank [54], authors observed the differences between individual perception of various types of food in participants characterized by various FN level and they stated that ratings by neophobics and neophilics differed but only for the unfamiliar foods, but not for familiar ones. The individuals of high FN level may be more pessimistic about their future experiences with new foods. It corresponds with reluctance to go to restaurants serving Asian ethnic cuisines and to learn to cook Vietnamese dishes which was observed in the studied group in the case of neophobic individuals. Moreover, neophobic individuals not only avoid novel foods, but they also more often, than neophilics, declare that they do not like them [12]. Therefore, it could be hypothesized, that the neophobic respondents rate unfamiliar foods as worse and they do it even after they tried them. Taking this into account, the fact that respondents received menu, labeled as menu of Vietnamese restaurant, may have caused their rejection of some dishes, even if they may have been interested in some of them or even have tried some of them. It may be confirmed by the observations that even after testing the new foods, respondents with high FN level are less willing to try novel foods than neophilics, as a single exposure does not eliminate completely the neophobics’ reluctance [54].

Sociodemographic characteristics may be within the determinants of food choices, food perceptions and FN [55]. The main sociodemographic variable, that was thoroughly tested in several studies, is gender. Male respondents are, in general, more neophobic than women [55,56,57]; however, the effect of gender on FN could vary depending on region [58]. Another sociodemographic characteristic that has strong influence on FN level is age of the respondent but the relationship between FN level and age is not linear. The results show that FN occurs mainly in childhood [38] and may decrease with age progression [59], but may also increase in older age [10]. Moreover, some studies [55,56,58] indicated that in adults, the low level of education could also be related to the high FN level. The latest study by Okumus et al. [57] indicated that even generations from the same country, may have impact on FN, as authors found that Generation Y (the generation born in the 1980s and 1990s), while compared with Generation Z (the generation born in the late 1990s and early 2000s), is more prone to experiencing new foods, which may also influence their FN level.

In the context of differences between Polish and Vietnamese cuisines [29,30], it should be mentioned that individuals who are exposed to different cultures, are expected to be less food neophobic. Therefore, it should be indicated that acceptance or rejection of a new food products depends not only on a personal preferences and familiarity with a food product, but it is also related to a complex psychological mechanism, specific behaviors and cultural aspects, including openness to various cultures [55].

Despite the fact, that the study presents some novel observations associated with the influence of FN on food choices in adults, some limitations of the study should be also listed. The presented study was conducted in a heterogenous group of adults with a higher share of women and it was conducted using one mock Vietnamese-style menu, so the observations should be verified for other menus, but also for other populations. The other limitation is associated with the fact, that the food consumption patterns, which are linked to FN, were not analyzed within this study. Moreover, the potential cultural bias was not deeply analyzed, so they should be included in the further studies. Last but not least, the further studies that include minorities should be conducted.

On the basis on the conducted study, some implications and recommendations for producers and public health purposes could be formulated. The knowledge about food neophobia and its effects may be an important element for food producers or restaurants with non-traditional foods and dishes, to achieve a competitive advantage, by improving familiarity with specific product or by using known ingredients in non-traditional dishes. Moreover, taking into account the fact, that not properly balanced diets resulting from rejecting some type of products are becoming a growing problem for developed societies, knowledge about food neophobia and its consequences may facilitate solving the problem. The education programs as well as food exposure conducted within sensory workshops could allow to familiarize with new food products and as a result to improve diversity of diet.

## 5. Conclusions

The key finding from the conducted research results from the fact that FN in young respondents may reduce the frequency of choosing dishes containing unfamiliar ingredients and; as a result, it may cause lower diversity of consumed dishes. Taking into account that not properly balanced diets resulting from rejecting some type of products are becoming a growing problem, the FN should be taken into account in the general public health policy.

## Figures and Tables

**Table 1:** The dishes included to the developed Vietnamese model restaurant menu with their descriptions and defined neophobic potential.

Course	Name of Dish (in Vietnamese)	Description of a Dish	Basis for Defining a Dish as One of Neophobic Potential for Polish Consumers
Starter	Nem quôń	Non-fried spring-rolls with shrimps, rice noodles, omelet, water cress and lettuce	Ingredients: shrimps, water cress Ingredients not typical for a specific dish: omelet in spring-rolls Preparation: non-fried spring-rolls
	Nem rán	Fried spring-rolls with turkey meat, rice noodles, mushrooms, turnip cabbage, carrot and mung bean sprouts	-
	Bánh gõi	Dumplings with pork meat and mushrooms	-
Soup	Phở gà	Soup of beef and poultry broth with meat, rice noodles, chive, mung bean sprouts and coriander	-
	Bún bò	Soup of pork and poultry broth with meat, rice noodles, chive and onion	-
	Mién gà	Soup of vegetable broth with boiled pork, cellophane noodles, nam huong mushrooms and chive	Ingredients: cellophane noodles, nam huong mushrooms
Main course	Cơm rang thịt gà	Fried rice with chicken, egg, carrot, pepper, peas and herbs	-
	Phở xào bò	Fried rice noodles with soy sauce, fish sauce, beef, green beans, mung bean sprouts and nuts	Ingredients: fish sauce Ingredients not typical for a specific dish: nuts in main course
	Bún chả lá lõt	Cooked rice noodles with grilled meat-stuffed cabbage with pork and beef cutlets	-
Dessert	Bánh gai	Pastry made of rice with lotus fruit, golden gram and coconut shreds	-
	Chè thập cãm	Dessert of golden gram, black eye beans, Azuki beans, tapioca, fruits, coconut milk and nuts, served in layers in a glass	Ingredients: black eye beans, Azuki beans, tapioca Preparation: served in layers in a glass
	Sữa chua vải	Lychee yoghurt with chia seeds and mango	-

**Table 2:** Characteristics of the participants of the study (*n* = 601).

Characteristics		*n* (%)
Age (years)	Mean ± SD	21.8 ± 3.2
	Median * (25th–75th)	21 (20–23)
Age (years)	18–21	346 (75.6)
	22–25	200 (33.3)
	>26	55 (9.2)
Sex	Women	425 (70.7)
	Men	176 (29.3)
Residence	Village	105 (17.5)
	Towns and cities of <500,000 residents	184 (30.6)
	Cities of >500,000 residents	312 (51.9)
Economic status	Very bad or bad	28 (4.7)
	Average	246 (40.9)
	Good or very good	310 (51.6)
	No answer	17 (2.8)

* Nonparametric distribution (for the Shapiro–Wilk test *p* ≤ 0.05).

**Table 3:** The FNS scores in the participants of the study (*n* = 601).

FNS Score	Total (*n* = 601)	Food Neophobia Level		
		Low (*n* = 200)	Average (*n* = 201)	High (*n* = 200)
Mean ± SD	31.0 ± 10.0	19.6 ± 4.1	31.3 ± 2.6	41.9 ± 5.3
95% CI	30.2–31.8	19.0–20.2	31.0–31.7	41.2–42.7
Median	31.0 *	20.0 *	31.0 *	41.0 *
25th–75th	23.0–28.0	17.0–23.0	28.0–34.0	38.0–44.5

FNS—Food Neophobia Scale; * nonparametric distribution (for the Shapiro–Wilk test *p* ≤ 0.05).

**Table 4:** The comparison of the general approach towards Vietnamese cuisine and towards information about allergens, accompanied by hunger sensation, stratified by food neophobia level of participants.

Items		Total (*n* = 601)	Food Neophobia Level			*p*-Value **
			Low (*n* = 200)	Average (*n* = 201)	High (*n* = 200)	
I would like to go to restaurants serving Asian ethnic cuisines	Mean±SD	5.3 ± 1.6	6.4 ± 0.9	5.3 ± 1.3	4.1 ± 1.5	<0.0001
	95% CI	5.1–5.4	6.2–6.5	5.1–5.5	3.9–4.4	
	Median	5.0 *	7.0 *^a^	5.0 *^b^	4.0 *^c^	
	25th–75th	4.0–7.0	6.0–7.0	5.0–6.0	3.0–5.0	
I would try a dish of not attractive appearance	Mean ± SD	3.5 ± 1.7	4.3 ± 1.7	3.5 ± 1.5	2.8 ± 1.6	<0.0001
	95% CI	3.4–3.7	4.1–4.5	3.2–3.7	2.6–3.0	
	Median	4.0 *	4.0 *^a^	3.0 *^b^	2.0 *^c^	
	25th–75th	2.0–5.0	3.0–5.0	2.0–5.0	2.0–4.0	
I would try a dish of unpleasant smell	Mean ± SD	2.0 ± 1.3	2.5 ± 1.5	2.0 ± 1.2	1.6 ± 1.0	<0.0001
	95% CI	1.9–2.2	2.3–2.7	1.8–2.1	1.5–1.8	
	Median	2.0 *	2.0 *^a^	2.0 *^b^	1.0 *^c^	
	25th–75th	1.0–3.0	1.0–3.0	1.0–2.0	1.0–2.0	
I would like to learn to cook Vietnamese dishes	Mean ± SD	4.5 ± 1.8	5.4 ± 1.5	4.5 ± 1.7	3.4 ± 1.6	<0.0001
	95% CI	4.3–4.6	5.2–5.7	4.3–4.8	3.2–3.6	
	Median	4.0 *	6.0 *^a^	5.0 *^b^	4.0 *^c^	
	25th–75th	3.0–6.0	4.0–7.0	4.0–6.0	2.0–4.0	
While ordering a dish I take into account information about allergens	Mean ± SD	2.0 ± 1.7	1.9 ± 1.7	2.0 ± 1.6	2.1 ± 1.8	0.2795
	95% CI	1.9–2.1	1.7–2.1	1.8–2.2	1.8–2.3	
	Median	1.0 *	1.0 *^a^	1.0 *^a^	1.0 *^a^	
	25th–75th	1.0–2.0	1.0–2.0	1.0–2.0	1.0–2.0	
I am hungry now	Mean ± SD	1.4 ± 0.5	1.5 ± 0.5	1.4 ± 0.5	1.4 ± 0.5	0.6293
	95% CI	1.4–1.5	1.4–1.5	1.3–1.5	1.4–1.5	
	Median	1.0 *	1.0 *	1.0 *	1.0 *	
	25th–75th	1.0–2.0	1.0–2.0	1.0–2.0	1.0–2.0	

* Nonparametric distribution (verified using Shapiro–Wilk test *p* ≤ 0.05); ** compared using chi^2^; ^a, b, c^ differing superscript letters indicate significance of differences between compared values.

**Table 5:** The comparison of the choices of dishes from the Vietnamese model restaurant menu, stratified by food neophobia level of participants.

Course	Dish	Total (*n* = 601)	Food Neophobia Level—*n* (%)			*p*-Value *
			Low (*n* = 200)	Average (*n* = 201)	High (*n* = 200)	
Starters	Nem quõn	126 (21.0)	62 (31.0)	47 (23.4)	17 (8.5)	0.0003
	Nem rán	220 (36.6)	79 (39.5)	72 (35.8)	69 (34.5)	
	Bánh gõi	187 (31.1)	56 (28.0)	66 (32.8)	65 (32.5)	
	I wouldn’t choose any	68 (11.3)	3 (1.5)	16 (8.0)	49 (24.5)	-
Soup	Phở gà	295 (49.1)	122 (61.0)	100 (49.8)	73 (36.5)	<0.0001
	Bún bò	129 (21.5)	17 (8.5)	50 (24.9)	62 (31.0)	
	Mién gà	104 (17.3)	49 (24.5)	29 (14.4)	26 (13.0)	
	I wouldn’t choose any	73 (12.1)	12 (6.0)	22 (10.9)	39 (19.5)	-
Main course	Cơm rang thịt gà	220 (36.6)	51 (25.5)	78 (38.8)	91 (45.5)	<0.0001
	Phở xào bò	158 (26.3)	87 (43.5)	46 (22.9)	25 (12.5)	
	Bún chả lá lõt	182 (30.3)	58 (29.0)	64 (31.8)	60 (30.0)	
	I wouldn’t choose any	41 (6.8)	4 (2.0)	13 (6.5)	24 (12.0)	-
Dessert	Bánh gai	143 (23.8)	59 (29.5)	54 (26.9)	30 (15.0)	0.0007
	Chè thập cãm	73 (12.1)	36 (18.0)	21 (10.4)	16 (8.0)	
	Sữa chua vải	307 (51.1)	89 (44.5)	108 (53.7)	110 (55.0)	
	I wouldn’t choose any	78 (13.0)	16 (8.0)	18 (9.0)	44 (22.0)	-

* Compared using chi^2^.

## Data Availability

The Data Availability Statement is not needed for the manuscript, as we did use neither available datasets, nor generated data for this study.
